# Association of serum bone- and muscle-derived factors with age, sex, body composition, and physical function in community-dwelling middle-aged and elderly adults: a cross-sectional study

**DOI:** 10.1186/s12891-019-2650-9

**Published:** 2019-06-05

**Authors:** Kenta Moriwaki, Hiromi Matsumoto, Shinji Tanishima, Chika Tanimura, Mari Osaki, Hideki Nagashima, Hiroshi Hagino

**Affiliations:** 1 Department of Orthopedic Surgery, Faculty of Medicine, Tottori University, Nishicho 36-1, Yonago, Tottori, 683-8504 Japan; 2 Department of Rehabilitation, Faculty of Health Science and Technology, Kawasaki University of Medical Welfare, Matsushima 288, Kurashiki, Okayama, 701-0193 Japan; 3 School of Health Science, Faculty of Medicine, Tottori University, Nishicho 86, Yonago, Tottori, 683-8504 Japan; 4 Rehabilitation Division, Tottori University Hospital, Nishicho 36-1, Yonago, Tottori, 683-8504 Japan

**Keywords:** Sclerostin, Insulin-like growth factor-1, Osteocalcin, Myostatin, Osteoporosis, Sarcopenia

## Abstract

**Background:**

Understanding interactions between bone and muscle based on endocrine factors may help elucidate the relationship between osteoporosis and sarcopenia. However, whether the abundance or activity of these endocrine factors is affected by age and sex or whether these factors play a causal role in bone and muscle formation and function is unclear. We aimed to evaluate the association of serum bone- and muscle-derived factors with age, sex, body composition, and physical function in community-dwelling middle-aged and elderly adults.

**Methods:**

In all, 254 residents (97 men, 157 women) participated in this cross-sectional study conducted in Japan. The calcaneal speed of sound (SOS) was evaluated by quantitative ultrasound examination. Skeletal muscle mass index (SMI) was calculated by bioelectrical impedance analysis. Grip strength was measured using a dynamometer. Gait speed was measured by optical-sensitive gait analysis. Serum sclerostin, osteocalcin (OC), insulin-like growth factor-1 (IGF-1), myostatin, and tartrate-resistant acid phosphatase-5b (TRACP-5b) concentrations were measured simultaneously. The difference by sex was determined using t test. Correlations between serum bone- and muscle-derived factors and age, BMI, SOS, SMI, grip strength, gait speed, and TRACP-5b in men and women were determined based on Pearson’s correlation coefficients. Multiple regression analysis was performed using the stepwise method.

**Results:**

There was no significant difference with regard to age between men (75.0 ± 8.9 years) and women (73.6 ± 8.1 years). Sclerostin was significantly higher in men than in women and tended to increase with age in men; it was significantly associated with SOS and TRACP-5b levels. OC was significantly higher in women than in men and was significantly associated with TRACP-5b levels and age. IGF-1 tended to decrease with age in both sexes and was significantly associated with SOS and body mass index. Myostatin did not correlate with any assessed variables.

**Conclusions:**

Sclerostin was significantly associated with sex, age, and bone metabolism, although there was no discernable relationship between serum sclerostin levels and muscle function. There was no obvious relationship between OC and muscle parameters. This study suggests that IGF-1 is an important modulator of muscle mass and function and bone metabolism in community-dwelling middle-aged and elderly adults.

## Background

Osteoporosis and sarcopenia are global problems that increase with longer life expectancy. Osteoporosis elevates the risk of fragility fractures and poor quality of life, and imposes a heavy economic burden [1]. Sarcopenia is characterized by a decline in muscle strength and mobility and an increase in falls and disability [2]. The prevalence of sarcopenia in Japanese women with osteoporosis was 29.7% in 2013 [3]. The risk of low bone mineral density (BMD) of the femoral neck and lumbar spine has been shown to increase significantly with sarcopenia in the Taiwanese population [4]. Therefore, understanding the interactions between bone and muscle may help elucidate the relationship between osteoporosis and sarcopenia.

Muscles interact with bones mechanically and functionally in skeletal tissues. Endocrine factors, as well as mechanical factors, may affect both muscle and bone metabolism. Sclerostin, a bone-derived factor, is an important negative regulator of bone formation and plays a key role in regulating the response to mechanical loading [5, 6]. Several previous studies have shown that men have significantly higher serum sclerostin concentrations than women and that serum sclerostin concentrations are positively correlated with age in healthy men [7, 8]. Serum sclerostin was positively correlated with BMD in the lumbar vertebrae and femur, as measured by dual energy X-ray absorptiometry (DXA), in postmenopausal women and with trabecular volumetric BMD, as measured by high-resolution peripheral quantitative computed tomography, in adult men [8, 9]. Sclerostin is an important negative regulator of bone formation that plays a key role in regulating the response to mechanical loading [5, 6]. Serum sclerostin was significantly lower in a group with high levels of physical activity than in a group with low levels of physical activity [7], whereas sclerostin was higher in patients with paralysis resulting from spinal cord injuries than in those with no injury [10].

Osteocalcin (OC), secreted by osteoblasts, is often used as a marker of bone formation [11]. A previous study demonstrated the relationship between bone metabolism markers and mechanical loading. OC and Insulin-like growth factor-1 (IGF-1) were significantly positively correlated with high-force eccentric exercise, and exercise caused an increase in OC and serum tartrate-resistant acid phosphatase-5b (TRACP-5b), which promotes increased bone metabolism [12].

IGF-1 is known for its physiological and pathological roles in the regulation of bone metabolism [13, 14]. Several studies suggest that IGF-1 is an important modulator of muscle mass and function, not only during the developmental period but also across the entire life span [15]. Serum IGF-1 concentrations are significantly lower in elderly women than in young women [16]. High serum IGF-1 concentrations are associated with the likelihood of being sarcopenic [17].

Myostatin is a highly conserved member of the transforming growth factor-β family functioning as a potent negative regulator of growth, and it is highly concentrated in skeletal muscles. Since the discovery of myostatin in skeletal muscles, there has been great interest in its role as a potential mediator of sarcopenia and as a therapeutic target [18]. Regarding serum myostatin levels, several reports have indicated different results; myostatin was reported to be significantly higher in men than in women and it negatively correlated with lean body and muscle mass [19, 20]. In contrast, in another study, myostatin was reported to positively correlate with muscle mass [21]. Further, age-related declines in bone mineral content and density were reported to be attenuated in myostatin-deficient mice [22].

Understanding the interactions between bone and muscle based on endocrine factors may help elucidate the relationship between osteoporosis and sarcopenia. However, it is unclear whether the abundance or activity of these endocrine factors is affected by age and sex or whether these factors play a causal role in bone and muscle metabolism. Thus, this study aimed to evaluate the association of serum bone- and muscle-derived factors with age, sex, body composition, and physical function in community-dwelling middle-aged and elderly adults.

## Methods

### Participants

This is a cross-sectional, population-based, observational study conducted in the town of Hino, Tottori Prefecture, Japan [2, 23–25]. In 2015, the town population comprised 3278 residents and approximately 47% of the residents were 65 years or older. Study participants were recruited from a pool of individuals who had registered for an annual town-sponsored medical check-up. A total of 1357 individuals aged 40 years or older were eligible to receive the annual town-sponsored medical check-up in 2016. Three individuals were excluded because it was certified that they required long-term care. A self-administered questionnaire was sent to 1354 participants. The inclusion criteria for participation in the study were as follows: (i) agreement to participate; (ii) living independently; and (iii) the ability to walk to the survey site and provide self-reported data. Accordingly, a total of 254 residents (97 men, 157 women) participated in the study.

### Characteristics of participants

Characteristics of participants such as age, sex, height, weight, and body mass index (BMI) were recorded during the medical check-up.

### Quantitative bone ultrasound imaging

Quantitative ultrasound (QUS) imaging was used to assess calcaneal bone mass. The measured calcaneal speed of sound (SOS) was evaluated using an ultrasound bone densitometer (CM-200; Furuno Electric Co., Nishinomiya, Japan) with a temperature correcting function. All participants placed their right heel on the quantitative ultrasound device while being seated. A coupling gel was applied to each participant’s right heel to facilitate the transmission of ultrasound waves to the skeletal site being examined.

### Muscle mass

Muscle mass was measured by bioelectrical impedance analysis (BIA) using a body composition analyzer (MC-780A; Tanita Co., Tokyo, Japan). The BIA method required participants to step onto a platform resembling a bathroom scale and remain in a standing position for approximately 30 s. Skeletal muscle mass index (SMI) was calculated by dividing limb muscle mass by height (kg/m^2^).

### Grip strength

Grip strength was measured using a dynamometer (T.K.K.5401; Takei Scientific Instruments Co., Niigata, Japan). Each participant was asked to squeeze the dynamometer twice with each hand. The highest score for each hand was recorded as the representative value.

### Gait speed

Gait speed was measured once for each participant by optical-sensitive gait analysis (Optogait; Microgate Co., Bolzano, Italy). Each participant was instructed to walk at their normal speed and the mean gait speed was calculated using a software program (Optogait analysis software, version 1.6.4.0; Microgate Co.) during 5 m of walking at a comfortable speed.

### Serum biochemical measurements

Blood samples were collected at the same time when parameters of body structure and physical function were measured. Samples were centrifuged for 15 min at 3000 r.p.m. at 4 °C and stored at − 80 °C until final analysis. Serum sclerostin levels were measured using an enzyme-linked immuno-sorbent assay (ELISA) kit (Sclerostin ELISA kit; BioMedica, Vienna, Austria). Assay buffer (150 μL/well) was added to 20 μL of standards, controls, and samples; this was followed by the addition of 50 μL of sclerostin antibody to each well. The plate was incubated for 24 h at 24 °C. Then, wells were washed five times, and 200 μL of conjugate was added. The plate was incubated in the dark for 1 h. Wells were washed, and 200 μL 3,3′,5,5′-tetramethylbenzidine was added to each well. Color was allowed to develop for 30 min at 24 °C, followed by the addition of 50 μL of stop solution. Absorbance was read at 450 nm within 10 min. Serum myostatin levels were measured using an ELISA kit (Myostatin ELISA kit; Immundiagnostik, Bensheim, Germany). Serum IGF-1 concentrations were measured by radioimmunoassay. Serum OC concentrations were measured by an electrochemiluminescence immunoassay. Serum TRACP-5b concentrations were measured by an enzyme immunoassay.

### Statistical analysis

The differences in age, height, weight, BMI, SOS, SMI, grip strength, gait speed, and sclerostin, OC, IGF-1, myostatin, and TRACP-5b levels between sexes were determined using the Student’s t test. Associations between serum bone and muscle-derived factors and age in men and women were determined based on Pearson’s correlation coefficients. Correlations between serum bone and muscle-derived factors and age, sex, BMI, SOS, SMI, grip strength, gait speed, and TRACP-5b levels were determined based on Pearson’s correlation coefficients. Multiple regression analysis was performed with serum sclerostin, OC, and IGF-1 as dependent variables and age, sex, BMI, SOS, SMI, grip strength, and gait speed as independent variables. Selection of regression models was done using the stepwise method. We judged multicollinearity by variance inflation factor (VIF). All data were analyzed using SPSS statistical software (version 24 for Windows; IBM Co., Tokyo, Japan). A *p*-value < 0.05 was considered significant.

## Results

### Demographics, parameters of body composition and physical function, and serum bone and muscle-derived factors

Demographics, parameters of body composition and physical function, and serum bone and muscle-derived factors for men and women and the results of the statistical comparison between them are shown in Table 1. There was no significant difference between men (75.0 ± 8.9 years) and women (73.6 ± 8.1 years) with regard to mean age (*p* = 0.198). BMI was significantly higher in men than in women (*p* = 0.014). Right calcaneal SOS, SMI, and grip strength were significantly higher in men than in women (*p* < 0.001). Sclerostin levels were significantly higher in men than in women (*p* < 0.001). OC concentration was significantly higher in women than in men (*p* < 0.001). TRACP-5b levels were significantly higher in women than in men (*p* = 0.026).

**Table 1:** Demographics, parameters of body composition and physical function, and serum bone- and muscle-derived factors

	Men (*n* = 97)	Women (*n* = 157)	*p* ^a^
Age (years)	75.0 ± 8.9	73.6 ± 8.1	0.198
Height (cm)	163.1 ± 6.5	150.0 ± 6.5	< 0.001
Weight (kg)	60.9 ± 8.0	49.5 ± 8.0	< 0.001
BMI (kg/m^2^)	23.0 ± 2.5	22.0 ± 3.1	0.014
SOS^b^ (m/s)	1503.6 ± 28.3	1483.4 ± 20.2	< 0.001
SMI (kg/m^2^)	7.5 ± 0.8	6.1 ± 0.8	< 0.001
Grip strength (kg)	34.9 ± 7.4	23.0 ± 4.5	< 0.001
Gait speed (m/s)	1.2 ± 0.3	1.3 ± 0.3	0.330
Sclerostin (pmol/L)	55.9 ± 26.1	32.6 ± 13.8	< 0.001
OC (ng/ml)	16.6 ± 5.5	21.5 ± 8.9	< 0.001
IGF-1 (ng/ml)	97.1 ± 30.5	93.4 ± 29.8	0.350
Myostatin (ng/ml)	42.9 ± 10.3	43.9 ± 12.2	0.495
TRACP-5b (mU/dL)	289.9 ± 95.4	340.3 ± 147.4	0.026

*BMI,* body mass index; *SMI,* skeletal muscle mass index; *SOS,* speed of sound; *OC,* osteocalcin; *IGF-1,* insulin-like growth factor-1*;TRACP-5b,* tartrate-resistant acid phosphatase-5b

All value are means ± SD

^a^ Difference between men and women (*t* test)

^b^ SOS of right calcaneus

### Association of serum bone and muscle-derived factors with age between sexes

**Fig. 1:**
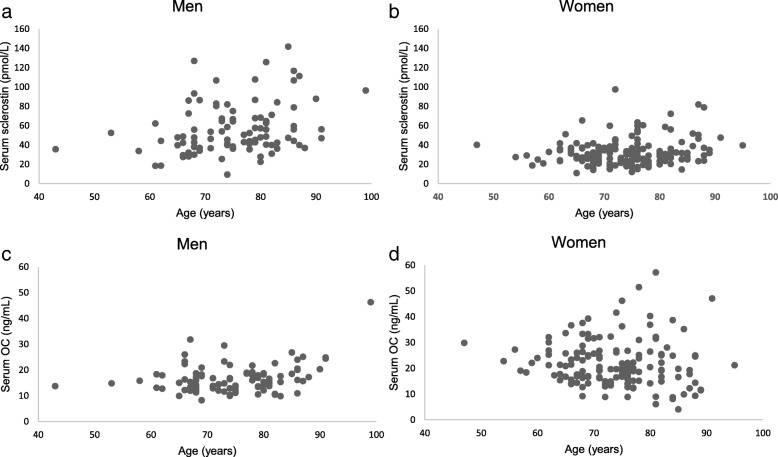
Associations of serum sclerostin with OC and age in both sexes. (a) Sclerostin is positively correlated with age in men (r = 0.318, *p* = 0.002). (b) Sclerostin is positively correlated with age in women (r = 0.180, *p* = 0.024). (c) OC is positively correlated with age in women (r = 0.314, p = 0.002). (d) OC does not correlate with age in men (r = − 0.075, *p* = 0.358)

**Fig. 2:**
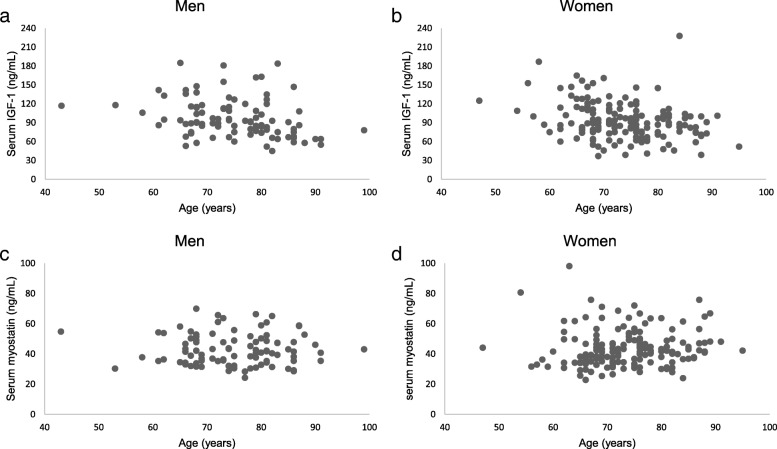
Associations of serum IGF-1 and myostatin with age in both sexes. (a) IGF-1 is negatively correlated with age in men (r = − 0.290, *p* < 0.001). (b) IGF-1 is negatively correlated with age in women (r = − 0.296, *p* = 0.003). (c) Myostatin does not correlate with age in men (r = 0.058, *p* = 0.474). (d) Myostatin does not correlate with age in women (r = − 0.029, *p* = 0.781)

Serum sclerostin and OC concentrations are shown by scatter plots in Fig. 1. Sclerostin was positively correlated with age in men (*r* = 0.318, *p* = 0.002) and women (r = 0.180, *p* = 0.024). OC was positively correlated with age in women (r = 0.314, p = 0.002) but not in men (r = − 0.075, *p* = 0.358). Serum IGF-1 and myostatin concentrations are shown in Fig. 2. IGF-1 was negatively correlated with age in men (*r* = − 0.290, *p* < 0.001) and women (*r* = − 0.296, *p* = 0.003). Serum myostatin was not correlated with age in both men (*r* = 0.058, *p* = 0.474) and women (r = − 0.029, *p* = 0.781).

### Correlations of serum bone and muscle-derived factors with demographics and parameters of body composition and physical function

Pearson’s correlation coefficients are shown in Table 2. Sclerostin was positively correlated with age, height, weight, BMI, SOS, SMI, and grip strength, and negatively correlated with sex, OC, TRACP-5b. OC was positively correlated with sex and TRACP-5b, and negatively correlated with height, weight, SOS, SMI, and grip strength. IGF-1 was positively correlated with height, weight, BMI, SOS, SMI, grip strength, and gait speed, and negatively correlated with age, OC, and TRACP-5b. Myostatin was not correlated with any of the assessed variables.

**Table 2:** Correlations of serum bone- and muscle-derived factors with demographics and parameters of body composition and physical function

	Age	Sex^a^	Height	Weight	BMI	SOS	SMI	Grip strength	Gait speed	Sclerostin	OC	IGF-1	Myostatin	TRACP-5b
Sclerostin	0.254^**^	−0.506^**^	0.323^**^	0.315^**^	0.149^*^	0.285^**^	0.356^**^	0.309^**^	−0.088	1	−0.188^**^	−0.027	0.073	−0.190^**^
OC	0.015	0.295^**^	−0.189^**^	−0.209^**^	−0.106	−0.260^**^	−0.250^**^	−0.255^**^	−0.098	−0.188^**^	1	−0.145^*^	−0.058	0.637^**^
IGF-1	−0.286^**^	−0.059	0.202^**^	0.273^**^	0.197^**^	0.228^**^	0.134^*^	0.229^**^	0.127^*^	−0.027	− 0.145^*^	1	0.027	−0.162^**^
Myostatin	0.023	0.043	−0.058	0.046	0.117	0.020	0.044	−0.020	−0.017	0.073	−0.058	0.027	1	−0.005

*BMI,* body mass index; *SMI,* skeletal muscle mass index; *SOS,* speed of sound; *OC,* osteocalcin; *IGF-1,* insulin-like growth factor-1.; *TRACP-5b,* tartrate-resistant acid phosphatase-5b

^*^p < 0.05, ^**^p < 0.01

^a^ Women = 0, Men = 1

### Multiple regression analysis

Multiple regression analysis results are shown in Table 3. Independent predictors for sclerostin were sex (partial regression coefficient [B] = 18.91, standardized partial regression coefficient [β] = 0.40, *p* < 0.001), age (B = 0.75, β = 0.28, *p* < 0.001), SOS (B = 0.14, β =0.15, *p* = 0.009), and TRACP-5b (B = − 0.02, β = − 0.11, *p* = 0.037). Independent predictors for osteocalcin were TRACP-5b (B = 0.03, β = 0.62, *p* < 0.001), sex (B = − 2.49, β = − 0.15, *p* = 0.002), and age (B = − 0.10, β = − 0.11, *p* = 0.026). Independent predictors for IGF-1 were age (B = − 0.86, β = − 0.24, *p* < 0.001), SOS (B = 0.19, β = 0.16, *p* = 0.010), and BMI (B = 1.58, β = 0.14, *p* = 0.018). There was no independent predictor for myostatin.

**Table 3:** Multiple regression analysis

model		B	SE(B)	β	t	95% CI	*p*	R^2^
(a) Sclerostin as dependent variable								
1	Sex^a^	24.14	2.56	0.52	9.40	(19.08~29.20)	< 0.001	0.267
2	Sex^a^	23.13	2.50	0.49	9.24	(18.20~28.06)	< 0.001	0.311
	Age	0.58	0.14	0.21	4.04	(0.30~0.86)	< 0.001	
3	Sex^a^	19.71	2.72	0.42	7.23	(14.34~25.08)	< 0.001	0.332
	Age	0.71	0.14	0.26	4.80	(0.42~1.01)	< 0.001	
	SOS	0.15	0.05	0.17	2.92	(0.50~0.25)	0.004	
4	Sex^a^	18.91	2.73	0.40	6.91	(13.52~24.29)	< 0.001	0.342
	Age	0.75	0.14	0.28	5.06	(0.46~1.05)	< 0.001	
	SOS	0.14	0.05	0.15	2.64	(0.03~0.24)	0.009	
	TRACP5b	−0.02	0.01	−0.11	−2.10	(−0.03~ − 0.001)	0.037	
(b) OC as dependent variable								
1	TRACP-5b	0.03	0.003	0.63	12.78	(0.03~0.04)	< 0.001	0.404
2	TRACP-5b	0.03	0.003	0.60	12.15	(0.03~0.04)	< 0.001	0.428
	Sex^a^	−2.73	0.81	−0.16	−3.36	(−4.33~ − 1.13)	0.001	
3	TRACP-5b	0.03	0.003	0.62	12.46	(0.03~0.04)	< 0.001	0.438
	Sex^a^	−2.49	0.81	−0.15	−3.06	(−4.09~ − 0.89)	0.002	
	Age	−0.10	0.04	−0.11	−2.23	(−0.19~ − 0.01)	0.026	
(c) IGF-1 as dependent variable								
1	Age	−1.03	0.22	−0.29	−4.68	(−1.47~ − 0.60)	< 0.001	0.080
2	Age	−0.88	0.22	−0.24	−3.93	(−1.32~ − 0.42)	< 0.001	0.107
	SOS	0.21	0.07	0.17	2.85	(0.06~0.35)	0.005	
3	Age	−0.86	0.22	−0.24	−3.87	(−1.30~ − 0.42)	< 0.001	0.124
	SOS	0.19	0.07	0.16	2.60	(0.04~0.33)	0.010	
	BMI	1.58	0.66	0.14	2.38	(0.27~2.89)	0.018	

*B,* partial regression coefficient; *SE*, standard error; *β,* standardized partial regression coefficient; *t*, t-ratio; *95%CI*, 95% confidence interval; *p*, p-value; *R*^*2*^, coefficient of determination

*BMI,* body mass index; *IGF-1,* insulin-like growth factor-1; *OC,* osteocalcin; *SMI,* skeletal muscle mass index; *SOS,* speed of sound; *TRACP-5b,* tartrate-resistant acid phosphatase-5b

Multiple regression analysis was performed with serum sclerostin, OC, and IGF-1 as dependent variables, and with age, gender, BMI, SOS, SMI, grip power, gait speed, and TRACP-5b as independent variables

Selection of modelling was done using stepwise method

^a^ Women = 0, Men = 1

## Discussion

In this study, we evaluated the association of sclerostin, OC, IGF-1, and myostatin with age, sex, BMI, SOS (as a measure of bone mass), SMI (as a measure of muscle mass), grip strength (as a measure of muscle strength), gait speed (as a measure of physical function), and TRACP-5b (as an indicator of bone metabolism).

Sclerostin concentrations were significantly higher in men, and SOS was an independent positive predictor of sclerostin. Although this result is paradoxical given that sclerostin is an inhibitor of bone formation, one possible explanation for this positive correlation between sclerostin and SOS is that bones of a high density are rich in osteocytes, which produce sclerostin [26].

Sclerostin is an important negative regulator of bone formation that plays a key role in regulating the response to mechanical loading [5, 6]. Immobilized patients have higher serum sclerostin concentrations, associated with reduced bone formation [27]. These findings may be related to the mechanical effects muscles have on bones. During this study, we investigated the relationship between sclerostin and muscle and physical function, and determined that there was no observable obvious relationship between them. This may be because of the difference between immobilized patients and healthy adults. The subjects included in this study were community-dwelling adults with fairly higher activities than immobilized patients. The subjects had sufficient mechanical loading to their bones prior to enrollment in the study, which may have resulted in a decline in the relationship between serum sclerostin concentrations and physical function.

In postmenopausal women immobilized after a stroke, sclerostin correlated negatively with bone formation markers and positively with resorption markers [27]. The results of bone turnover markers suggest that there exists an imbalance between bone resorption and bone formation in immobilized patients [27]. Conversely, sclerostin correlated negatively with TRACP-5b because the bone resorption rate was high in healthy community-dwelling adults in this study.

OC concentrations were significantly higher in women than in men, and were significantly associated with TRACP-5b. OC concentrations were significantly positively correlated with high force eccentric exercise, which promotes increased bone metabolism [12]. In this study, we investigated the relationship between OC and muscle parameters, and determined that there was no observable obvious relationship between them.

IGF-1 is known to have an anabolic effect on bone [14]. IGF-1 has been reported to be positively correlated with BMD in elderly women, suggesting a correlation with bone formation [28]. We found that IGF-1 was significantly positively correlated with SOS, suggesting that IGF-1 plays an important role in bone formation. Increased IGF-1 expression with muscle hypertrophy also likely increases IGF-1 secretion and the local abundance of IGF-1 at the muscle-bone interface. Muscle hypertrophy and bone anabolism are connected through an IGF-1 mediated paracrine signaling mechanism [29]. Serum IGF-1 is useful for estimating the prevalence of vertebral fractures in patients with type 2 diabetes mellitus considering that decreased serum IGF-1 may be involved in the deterioration of bone quality [30].

IGF-1 and myostatin, both of which are myokines, mediate crosstalk among myocytes and are thought to be involved in the synthesis and decomposition of muscle proteins [31]. However, no consensus on the involvement of myostatin has been reached at this time. In this study, myostatin was not correlated with any of the assessed variables. One of possible reasons for this could be that it is difficult to distinguish between the active and inactive forms of myostatin using conventional quantification methods [18].

Limitations of our study are the relatively small sample size and the cross-sectional study design. The strength of this study is that it is the first to evaluate the association of serum bone- and muscle-derived factors with body composition and physical function simultaneously. In the future, it will be necessary to conduct longitudinal studies to confirm the findings of this study. These studies may eventually help elucidate the relationship between osteoporosis and sarcopenia.

## Conclusions

We found that sclerostin was significantly associated with sex, age, and bone, although there was no discernable relationship between serum sclerostin and muscle function. There was no obvious relationship between OC and muscle parameters. This study suggests that IGF-1 is an important modulator of not only muscle mass and function but also of bone in community-dwelling middle-aged and elderly adults.

## Data Availability

The datasets generated and analyzed during the current study are not publicly available because of professional discretion, as they were part of patient’s records, but are available as a de-identified data sheet from the corresponding author on reasonable request.

## Abbreviations

BIA: Bioelectrical impedance analysis
BMD: Bone mineral density
BMI: Body mass index
DXA: Dual energy x-ray absorptiometry
ELISA: Enzyme-linked immuno-sorbent assay
IGF-1: Insulin-like growth factor-1
OC: Osteocalcin
QUS: Quantitative ultrasound
SMI: Skeletal muscle mass index
SOS: Speed of sound
TRACP-5b: Tartrate-resistant acid phosphatase-5b
